# Stability in Vitiligo: Why Such a Hullabaloo?

**DOI:** 10.4103/0974-2077.53101

**Published:** 2009

**Authors:** Somesh Gupta

**Affiliations:** *Department of Dermatology and Venereology, Association of Cutaneous Surgeons of India (ACSI), All India Institute of Medical Sciences, New Delhi, India*

Questions have been raised about the stability in vitiligo for the past several years. However, during the same period several large series have been published showing successful melanocyte transplantation outcomes. In spite of repeated expression of concerns about the criteria determining the stability, several thousand patients have undergone transplantation successfully and benefited from these procedures. This by itself should be convincing enough, even to the skeptics and it seems that the debate on whether the ‘stability’ exists is more a theoretical concern than having much practical implications. We have shown that half the patients with recalcitrant bilateral vitiligo vulgaris and nearly 90% of those with segmental vitiligo with one year of stability, as determined by history, show >75% repigmentation at six months.[1] For most of the medical therapies, including narrow band ultraviolet B (NBUVB) and psoralen photochemotherapy (PUVA), these statistics would be considered as excellent. So then why so much debate, so much hue and cry?

Simple methods such as comparison of pictures taken six months apart and careful history will be largely adequate to determine stability in most of the cases. Careful examination may also give additional clues regarding stability. In progressive vitiligo, often the margins are ill-defined or show different shades of colors. One should refrain from performing surgeries in patients with large surface area involvements as such patients are unlikely to respond regardless of clinical and/or experimental stability.

There are several theories of the pathogenesis of vitiligo—biochemical, immunological, genetic and other biological aspects. Vitiligo is now considered as a complex reaction pattern or a syndrome, involving multiple etiologic factors.[2] Thus it is unlikely that there would be a single factor determining the stability. Several factors may play a role in disease activity or stability [Table 1].

**Table 1:** Potential objective markers of stability

**Biochemical**
Serum and tissue levels of catalase, glutathione peroxidase, superoxide dismutase
Plasma and urinary levels of catecholamines with a highly sensitive method such as high-pressure liquid chromatography (Nor-epinephrine, Epinephrine, and Dopamine)
Immunological
Immunohistochemistry of lesional biopsy. Number of cytotoxic
T lymphocytes, LFA-1 positive cells, and CD45 RO memory T cells and reversal of CD4/CD8 ratio
Genetic

We are investigating some of these factors. There are two main biochemical abnormalities associated with vitiligo. These include disturbed antioxidant defense as evidenced by high levels of hydrogen peroxide in the epidermis and elevated serum and urine levels of catacholamines. The latter is thought to be a result of the former. Catecholamines are a better substrate for tyrosinase than tyrosine. This causes production of o-quinones resulting in either heptanation of tyrosinase or generation of reactive oxygen species (ROS) triggering or aggravating melanocyte damage.[3] In our previous study, we have already shown the involvement of oxidative stress in vitiligo patients.[4] The levels of catecholamines, which are consistently released as a consequence of emotional and/or stressful events, are considered as being strictly related to the onset or worsening of the disease.[5]

Oxidative stress is proposed as a triggering event in melanocyte degeneration. Imbalance in the oxidant–antioxidant system leads to generation of reactive free radicals. Reactive free radicals bring about lipid peroxidation producing lipid peroxides and lipoxides whose further decomposition yields malondialdehyde, which causes damage to cell membrane or DNA leading to cytotoxicity, mutagenicity and cell death. Antioxidants scavenging them include catalase, glutathione peroxidase, and superoxide dismutase, which are potential markers of stability in vitiligo.

Although auto-antibodies to melanocytes have been identified in patients with active vitiligo, at present there is a preponderance of studies in support of cell-mediated auto-immunity in vitiligo.[6] So studying immunohistochemical markers of immune activation in the biopsy from the lesion before selecting for transplantation has a potential for determining the stability. A study though with a small sample size, has shown that after transplantation, repigmenting vitiligo lesions show significantly less number of cytotoxic T lymphocytes and LFA-1 positive cells than those not responding to transplantation.[7] There is a possibility of increase in CD45 RO memory T cells and reversal of CD4/CD8 ratio in active lesions of vitiligo.[7]

So in summary, while better objective markers are needed to define stability, we should condrutinue to rely on existing recommendations for stability[8] till such time we get more sensitive and specific objective criteria for patient selection and stability. With present expert recommendation on patient selection and stability, the majority of patients will be benefit from transplantation surgeries.
